# Effects of providing manuscript editing through a combination of in-house and external editing services in an academic hospital

**DOI:** 10.1371/journal.pone.0219567

**Published:** 2019-07-09

**Authors:** Joon Seo Lim, Vanessa Topping, Ji Sung Lee, Keenan D. Bailey, Sung-Han Kim, Tae Won Kim

**Affiliations:** 1 Scientific Publications Team, Clinical Research Center, Asan Institute for Life Sciences, Asan Medical Center, University of Ulsan College of Medicine, Seoul, Korea; 2 Department of Clinical Epidemiology and Biostatistics, Asan Medical Center, University of Ulsan College of Medicine, Seoul, Korea; 3 Clinical Research Center, Asan Institute for Life Sciences, Asan Medical Center, University of Ulsan College of Medicine, Seoul, Korea; 4 Department of Infectious Diseases, Asan Medical Center, University of Ulsan College of Medicine, Seoul, Korea; 5 Department of Oncology, Asan Medical Center, University of Ulsan College of Medicine, Seoul, Korea; Cedars-Sinai Medical Center, UNITED STATES

## Abstract

**Background:**

English editing services are effective for improving manuscript quality as well as providing learning opportunities for non-native English-speaking authors. Herein, we describe the effects of a combined system of in-house and external editing services for handling large volumes of editing requests and providing personalized editing service in academic hospitals.

**Methods:**

We established the Scientific Publications Team (SPT), an in-house editing team in Asan Medical Center in Seoul, Korea. The SPT is composed of two professional editors who manage editing requests sent to external companies while also providing one-on-one in-house editing services. We gathered author satisfaction data from 936 surveys between July 2017 and December 2018 and analyzed the number of editing requests and research publications by segmented regression analysis of interrupted time series data.

**Results:**

The SPT processed 3931 editing requests in 2017–2018, which was a marked increase compared with prior to its establishment (*P* = 0.0097). The authors were generally satisfied with the quality of editing services from both in-house and external editors. Upon conducting regular quality control, overall author satisfaction with one external company gradually increased over the course of one year (*P* for trend = 0.086). Author satisfaction survey results revealed that overall satisfaction of editing service was most strongly correlated with how well the edits conformed to the authors’ intentions (R = 0.796), and was only weakly correlated with quick turnaround time (R = 0.355). We also observed a significant increase in the trend of the number of research publications (*P* = 0.0007) at one year after the establishment of the SPT.

**Conclusion:**

Providing a combination of in-house and external editing services resulted in high author satisfaction and subsequent hospital-wide increases in manuscript writing and publication. Our model system may be adapted in academic hospitals to better address the editing needs of non-native English-speaking researchers.

## Introduction

Writing effective and persuasive research articles requires a wide range of communication skills such as language comprehension, data presentation, and establishment of logical flow, which poses difficulty even for native English-speaking researchers. Non-native researchers naturally encounter more challenges when writing manuscripts in English, which are compounded by the increasing pressure to “publish-or-perish” in academia. Such pressure is especially intense for physicians working in academic hospitals in Korea, where more emphasis tends to be placed on the quantity of research outputs rather than clinical hours for career advancement.

In academic hospitals, past generations of medical residents and fellows were able to receive informal tips-and-tools type education on manuscript writing from advisors within their respective departments. However, such mentor-mentee relationships are dissipating due to increased clinical workloads for professors and fellows alike [1,2], leaving early-career researchers lacking in terms of proper training in manuscript writing. Thus, Korean researchers have become increasingly burdened as they are faced with the task of writing scientific manuscripts in English.

With the English language having been established as the universal language of science [3], there is an urgent and critical need for quality English editing for scientific manuscripts written by non-native English speakers. As such, Asan Medical Center (AMC) in Seoul, Korea started providing a referral system to assist its researchers in submitting their manuscripts to external editing companies (EECs) in January 2011. While AMC researchers were generally satisfied with the service, there was a need to further improve author satisfaction by conducting regular in-house quality control of the external companies by professional editors who are experienced in scientific manuscript editing.

Additionally, the AMC authors expressed the need for personalized guidance from manuscript editors so as to ask detailed questions on specific English expressions or their manuscript as a whole. Most EECs offer an option for the authors to contact the editors by mediating e-mail communications or through on-line message board systems. However, it is often difficult to convey one’s intentions through writing only, especially when communicating in a foreign language. We reasoned that providing one-on-one consultation manuscript consultations may be effective in filling the communication gap between authors and editors.

Here, we introduce an implementable system for providing quality manuscript editing service through an in-house editing team that manages and controls the quality of the out-going editing requests as well as provides personalized in-person editing services. We describe the manuscript editing workflow that was used for handling > 2000 yearly editing requests and provide discussion on an effective mode of controlling the quality of external editing services through author satisfaction surveys. We also show the correlations between the establishment of the in-house editing team and the trends in the numbers of editing requests and research publications from our medical center.

## Materials and methods

### Study site selection

AMC was selected as the study site considering its high volume of research activity and publication. AMC is the largest tertiary care center in Korea, and its researchers publish on average > 1500 scientific papers per year; as such, the need for English editing is also high, which is necessary for gathering a large volume of survey responses.

### Manuscript editing request count

Researchers at AMC may fill out the “English editing request form” available on the hospital intranet to request English editing for research-related documents, including drafts of original manuscripts, revised manuscripts and response letters to reviewer comments, conference abstracts, and scripts for oral presentations, the last of which were exclusively handled by the in-house editors (S1 Table). We counted the total number of editing requests from the AMC intranet database and summarized the data by quarter. We also counted the number of editing requests that specifically requested an in-house editor.

### Author satisfaction survey

We designed the author satisfaction survey form using the Google Forms application (Google Inc., Mountain View, CA, USA), which comprised five questions on 5- or 7-point Likert scales and two yes-or-no questions (Table 1). Items #2, #3, and #7 were graded on 5-point Likert scales from July 2017 to January 2018, and were changed to 7-point scales in February 2018 in order to obtain more accurate measurement of author satisfaction; as such, 5-scale scores of those items were converted to 7-point scales in the final analysis by using a previously reported method [4].

**Table 1:** Questions and scoring system of the author satisfaction survey.

#	QUESTIONS	SCORING SYSTEM
**1**	**[Timeliness]** Was the request finished in a timely manner?	**5** = Very fast **1** = Very slow
**2**	**[Understanding]** How well did the editor understand your manuscript?	**7** = Understood very well **1** = Did not understand at all
**3**	**[Intentions]** Did the corrections conform with your original intentions?	**7** = Conformed very well **1** = Did not conform at all
**4**	**[Corrections]** How many corrections did you have to make afterwards?	**5** = None **4** = 1 to 2 **3** = 3 to 5 **2** = 6 to 10 **1** = Many
**5**	**[Re**-**request]** Are you willing to use this company’s editing service again?	Yes / No
**6**	**[Recommend]** Are you willing to recommend this team to your peers?	Yes / No
**7**	**[Overall]** Overall satisfaction rating of the editing service of this company	**7** = Very satisfied **1** = Very dissatisfied

From July 2017 to December 2018, we sent out the author satisfaction survey forms to the corresponding authors of 1709 manuscripts that were processed by the SPT. The surveys were divided into blind and non-blind surveys: for blind surveys, the identity of the editor or the editing company was hidden in the final edited file and the editing certificates were provided to the authors only after filling out the blind survey; for non-blind surveys, we sent the editing certificates to the authors along with the edited file, thereby informing the authors of the editing company involved. Non-blind surveys were sent out for cases in which hiding the identity of the editor was not possible, such as when authors asked for a specific editor/company or when a one-on-one consultation was carried out by the in-house editors. All other editing requests were randomly distributed with a 1:1 ratio of blind and non-blind surveys so that the results could be compared afterward to determine the magnitude of bias arising from the non-blinded satisfaction survey.

We only gathered survey responses from the requests for drafts of original studies from all major areas of clinical medicine as well as biomedical research. The survey forms were sent three business days after the authors received the edited manuscripts; this was intended to allow enough time for the researchers to review the edited manuscripts, as well as to prevent them from prematurely finishing the survey before fully reviewing the edits. The study protocol was approved by the institutional review board of AMC.

### Collection of data on research publications

We used the bibliographic database EMBASE to search for articles written by AMC-affiliated authors. To include all publications from the AMC campus, we used the following terms as the search string: “Asan Medical Center,” “University of Ulsan College of Medicine,” and “Asan Institute for Life Sciences.” The publication dates of the articles ranged from January 1^st^, 2011 to December 31^st^, 2018; the start date of the search period was chosen as January 1^st^, 2011 because AMC started providing English editing via the EECs at that timepoint. After downloading the search results, we selected the articles that listed AMC-affiliated authors as the corresponding author. The total number of articles were counted and summarized by quarter.

### Statistical analysis

In all analysis, statistical significance of *P* values was designated at 0.05. The survey responses were converted to 7-point scales and 5-point scales as appropriate; for analysis, the mean values of items #1, #2, #3, #4, and #7 were calculated, and in cases of Yes/No questions (items #5 and #6), the proportion of “Yes” responses were calculated. To delineate significant differences between groups in author satisfaction survey results, we performed Mann-Whitney *U* tests in GraphPad Prism version 7.00 for Windows (GraphPad Software, La Jolla, CA, USA). Differences between the survey response rates and the proportions of unsatisfactory responses among the groups were evaluated with the Chi-square test and the Fischer’s exact test, respectively.

To evaluate the effect of SPT establishment on the trends in the numbers of editing requests and research publications, we used SAS version 9.4 (SAS Institute Inc., Cary, NC, USA) to perform a segmented regression analysis of interrupted time series (ITS) data, which is a valuable study design for evaluating the effectiveness of population-level interventions that have been implemented at a clearly defined point in time [5–7]. To analyze the trend in the number of editing requests, January 2017 (introduction of the SPT) was chosen as the timepoint of the interruption, and January 2018 (one year after the introduction of the SPT) was chosen for trend in the number of publications. In the ITS analysis, the time series of interest was analyzed as the outcome measure with dummy variables entered as model predictors to indicate the pre- and post-interruption or intervention phases of the series following a segmented regression approach. The linear regression model (identity link function) for time series data was used. The single-ITS analysis (SITSA) model used the following regression:

$$\mathrm{Equation}:\;y=\alpha +\beta_{1}T+\beta_{2}X+\beta_{3}XT+\epsilon$$,

where y = outcome variable, α = intercept, β = coefficients, T = time (1, 2, 3, …, N), X = study phase (0 during pre-interruption and 1 during post-interruption), XT = time after interruption (0 during pre-interruption and 1, 2, 3, …, N during post-interruption), and ε = error or residual. The coefficient (β) for T indicates the pre-interruption slope. The primary coefficients of interest are β2 and β3 (for X and XT) which respectively indicate the change in level from pre- to post-interruption and the change in slope from pre- to post-interruption. The post-interruption slope was determined by summing the coefficients β1 and β3 with statistical significance obtained using post-estimation procedures. If an interruption or intervention has an effect on the outcome, then a change in level and/or slope should be detected between pre- and post- phases after accounting for any secular trend in the pre-interruption period.

## Results

### Establishment of the in-house editing team and the manuscript editing workflow

We established the Scientific Publications Team (SPT), an in-house manuscript editing team at AMC in January 2017. We made a hospital-wide announcement regarding the establishment of the SPT by using intranet mail and posters in each clinical department. The SPT is composed of a non-native bilingual (Korean and English) editor and a native English-speaking editor, both of whom had advanced degrees in biomedical research. The primary roles of the in-house editors include managing all editing requests, performing quality control for external editing companies, providing English editing for research-related documents and, importantly, offering one-on-one consultation with authors. The in-house editors undertook approximately 15% of the total editing requests, and the rest were sent out to four different EECs. The details of the EECs are presented in S2 Table.

**Fig 1:**
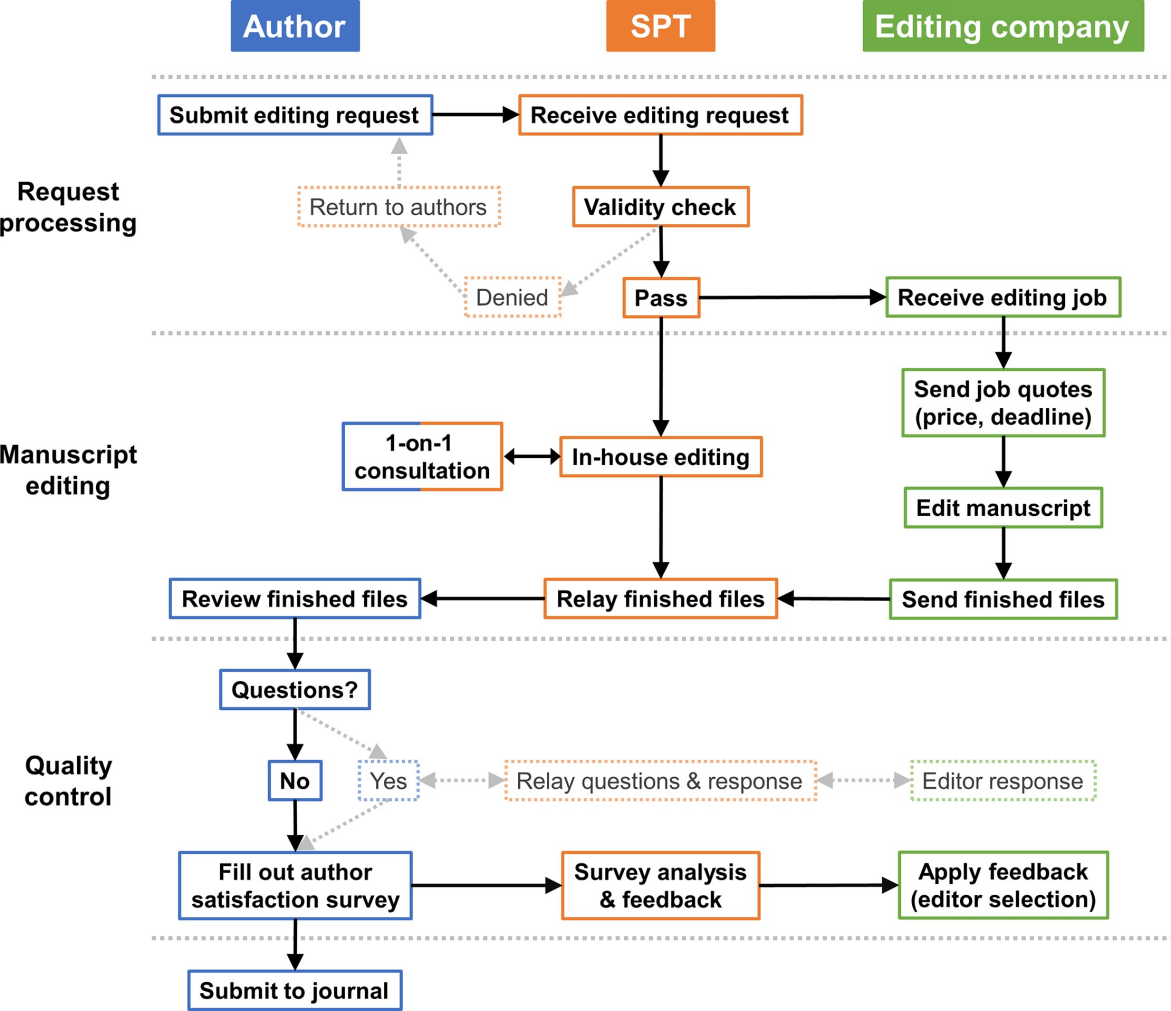
Manuscript editing workflow. Boxes with bold texts indicate the routine workflow in processing editing requests. Boxes in faded colors indicate processes that are infrequently carried out.

The overall workflow of the manuscript editing system is shown in Fig 1. Upon receiving editing requests from the authors, the SPT reviewed each request for validity and returned inappropriate requests. The validity check included the following questions: (1) Did the request receive final approval from the corresponding author? (2) Is a staff member of AMC (assistant professor or higher) included as either the first author or the corresponding author? (3) Is the document within the scope of the editing service (draft of research manuscript, revised manuscript, response to reviewer comments, abstract or oral presentation script for international conference, peer review comment)? (4) Is the manuscript targeted for submission to a SCI or SCI-E journal? After passing the validity check, the requests were randomly distributed among the EECs and the in-house editors unless the authors asked for a specific editing company or an in-house editor.

The SPT sent the editing requests to EECs via e-mail, which would then send job quotes that included the price and expected deadline. When requested, the in-house editors provided one-on-one consultation on the manuscripts either in-person, via phone, or e-mail. Such process of author-editor interaction was more complicated with the EEC, with the SPT and the EECs working as intermediaries between authors and editors (“Quality Control” panel in Fig 1). The final edited files from either EECs or the SPT were sent to the authors via e-mail.

Importantly, the SPT put emphasis on receiving detailed feedback on the editorial work of the EECs and the in-house editors by using the author satisfaction survey form (Table 1). Whenever negative feedback (e.g., scores 1–3 on a 7-point scale) was noted, the in-house editors contacted the authors to obtain more details regarding their dissatisfaction with the editing service, and reviewed the issue before addressing it with the EECs. As a result, editors at the EECs who received negative feedback were removed from our editing process, and those who received particularly positive feedback were placed on a list of preferred editors for future requests.

### Changes in the number of manuscript editing requests

**Fig 2:**
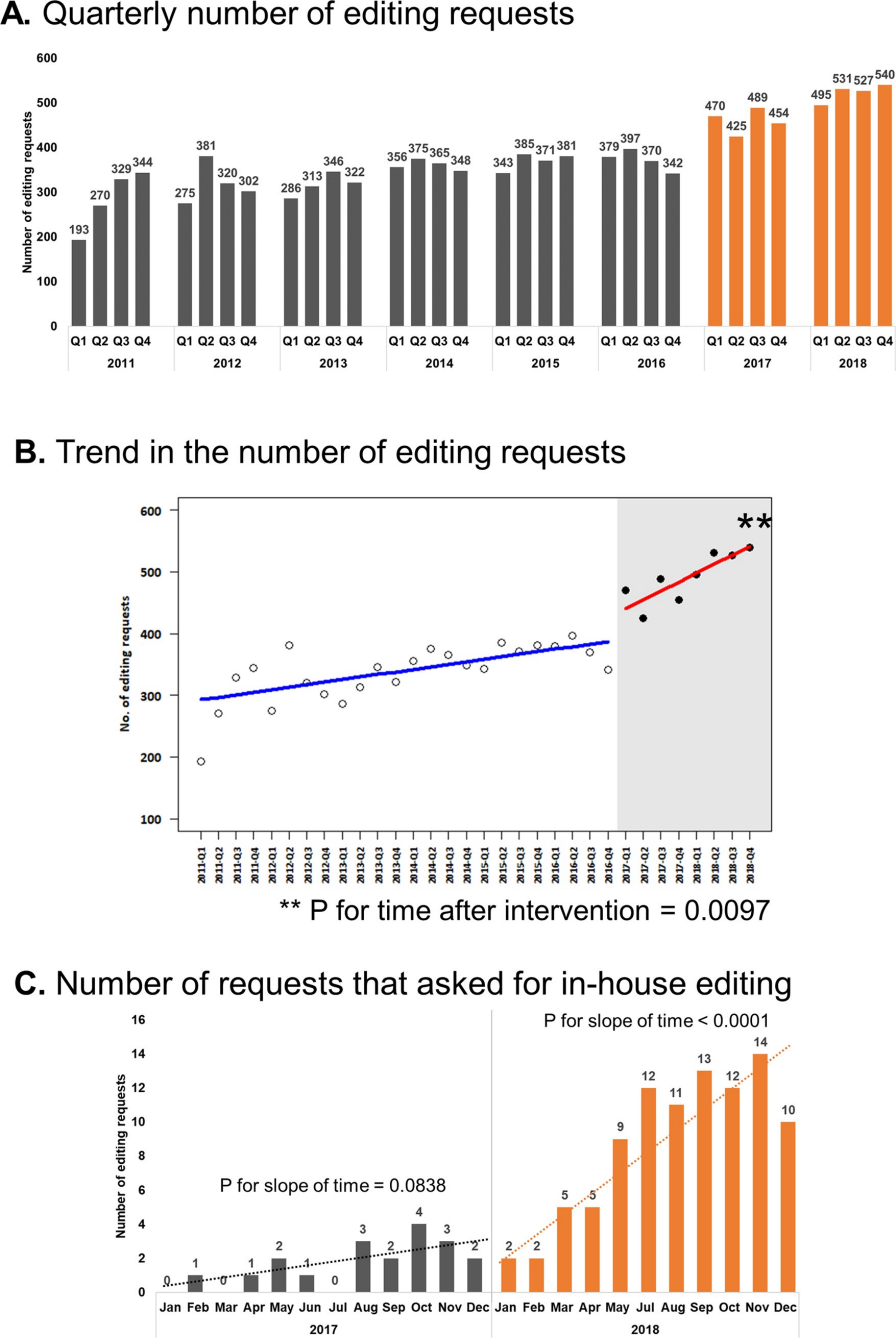
Trend in the number of editing requests. Trend of editing requests from AMC researchers from 2011 Q1 to 2018 Q4. (A) Quarterly number of editing requests. Numbers on top of the bars indicate the actual number of editing requests made during the respective quarters. Bars in orange indicate the period after which the Scientific Publications Team was established. (B) Trend in the number of editing requests. Asterisks (**) indicates a significant (*P* = 0.0097) difference in the slope of the editing request trend after intervention (establishment of the Scientific Publications Team; January 2017). (C) Number of requests that asked for in-house editing. Numbers on top of the bars indicate the actual number of editing requests that specifically asked for one of the in-house editors. Data from June 2018 was omitted because the in-house editors were on leave for most of the period, during which the number of editing requests was 2.

The establishment of the SPT was followed by a significant increase in the number of editing requests from AMC researchers. From 2011 to 2013, the average number of quarterly editing requests steadily increased from 284 to 317, and the number plateaued after 2014 at approximately 368 per quarter. After the SPT was launched, the average number of quarterly editing requests showed step-wise increases to 459.5 in 2017 and to 523.3 in 2018 (Fig 2A). Collectively, the SPT successfully processed a total of 1838 editing requests in 2017 and 2093 requests in 2018.

We performed a segmented regression analysis of interrupted time series data to compare the trend in the number of editing requests; by regarding the establishment of the SPT as a type of intervention, we observed a significant difference (*P* for time after intervention = 0.0097) in the number of editing requests after the launch of the SPT in 2017 (Fig 2B). We also analyzed the number of specific requests for in-house editing, and observed that such requests significantly increased in 2018 (*P* for slope of time < 0.0001) (Fig 2C).

### Author satisfaction survey–Overall satisfaction score

**Fig 3:**
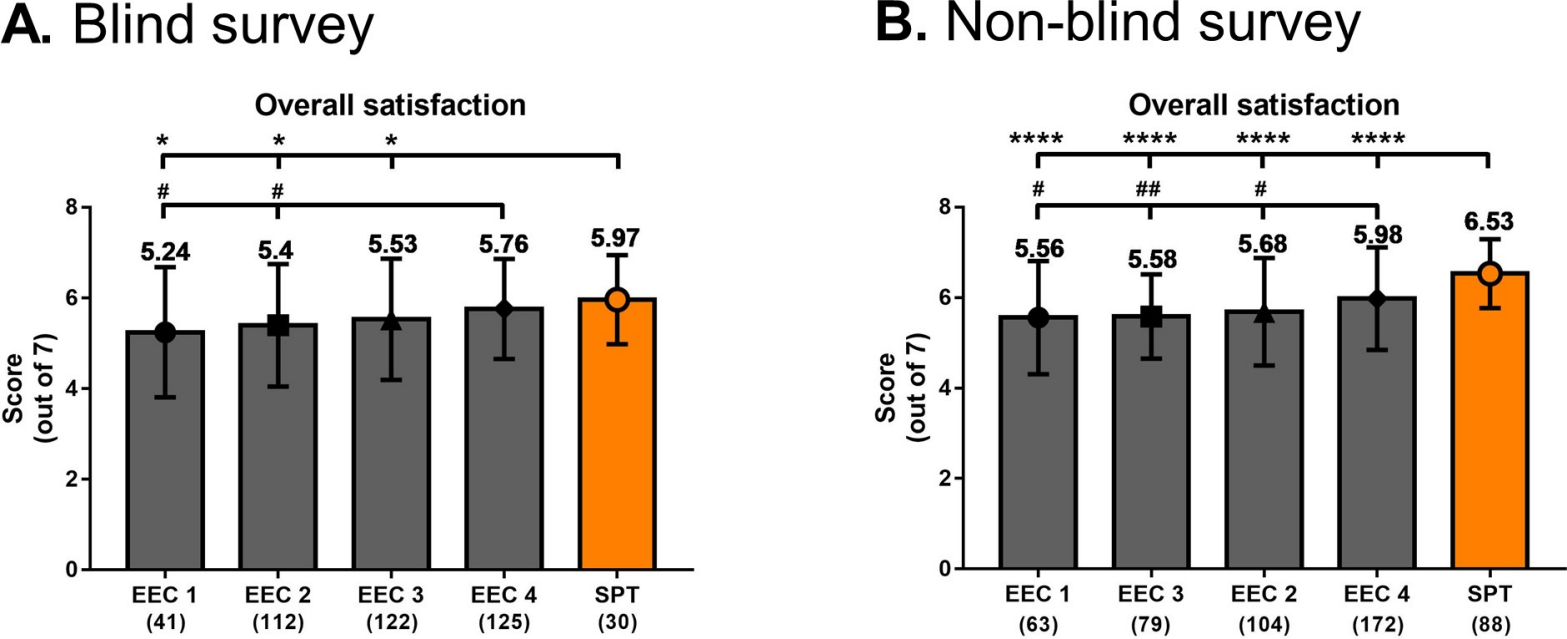
Overall satisfaction scores. Overall satisfaction scores of the EECs and the SPT from author satisfaction surveys gathered from July 2017 to December 2018. Overall satisfaction scores from (A) blind surveys and (B) non-blind surveys. Numbers on top of the bars indicate the mean values, and numbers in parentheses indicate the number of survey results. Error bars indicate standard deviation from mean. * *P* < 0.05 vs. SPT, # *P* < 0.05 vs. EEC 4; ## *P* < 0.01 vs. EEC 4; **** *P* < 0.0001 vs. SPT. EEC, external editing company; SPT, Scientific Publications Team.

A total of 936 survey responses (430 blind, 506 non-blind) were gathered; the overall response rates were 56.7% (430/758) and 53.2% (506/951) for blind and non-blind surveys, respectively, and no significant differences were observed among the five groups (4 EECs and the SPT) in terms of response rate (S3 Table). The authors were generally satisfied by the editorial works of the EECs and the in-house editors of the SPT; the overall satisfaction scores in the blind and non-blind surveys ranged from 5.24 to 5.97 and 5.56 to 6.53 out of 7, respectively (Fig 3).

Out of the 430 blind survey responses, 93 (21.6%) had originally been collected in 5-point scales and were later converted to 7-point scales prior to addition in the final analysis. We therefore compared the mean overall satisfaction scores of Fig 3A (7-point scale scores plus 5-point scale scores) and those of 7-point scale scores only in order to determine whether the addition of converted 5-point scale scores significantly affected the survey results (S4 Table). We first observed that the order of the EECs and the SPT in terms of mean overall satisfaction score remained the same when only the 7-point scale scores were used, with EEC 1 and SPT having the lowest and highest scores, respectively. More importantly, there were no significant differences within each company or the editing service providers as a whole in terms of overall satisfaction score between 7-point + 5-point scores and 7-point scores only (S4 Table), signifying that the addition of the converted 5-point scores did not significantly affect the overall satisfaction scores.

The results of the blind and non-blind surveys revealed similar patterns, with EEC 4 having the highest mean score among the EECs, and the SPT having the highest score overall. As expected, the overall satisfaction scores of all groups were higher in the non-blind surveys (Fig 3B) than in the blind surveys (Fig 3A); the differences in the scores between non-blind surveys and blind surveys in each company were as follows: 0.32, 0.28, 0.05, 0.22, and 0.56 in EEC 1, 2, 3, 4, and SPT, respectively. Notably, the overall satisfaction score of the SPT was significantly higher than those of all four EECs in the non-blind survey, and significantly higher than those of three EECs in blind survey.

### Comparison between survey responses from random and specific assignments

We ran a subgroup analysis of the non-blind survey results between randomly assigned requests and those that had requested for specific editors or companies (S5 Table). All non-blind survey results for the SPT were from specific requests and thus was not used in this comparison. In the EECs, 31.1% (130 of 288) of the responses were from specific requests. Within each company and the EECs as a whole, there were no significant differences between random and specific assignments in terms of overall satisfaction scores, albeit the scores in the “Specific” groups tended to be numerically higher.

### Author satisfaction survey–Specific questions

**Fig 4:**
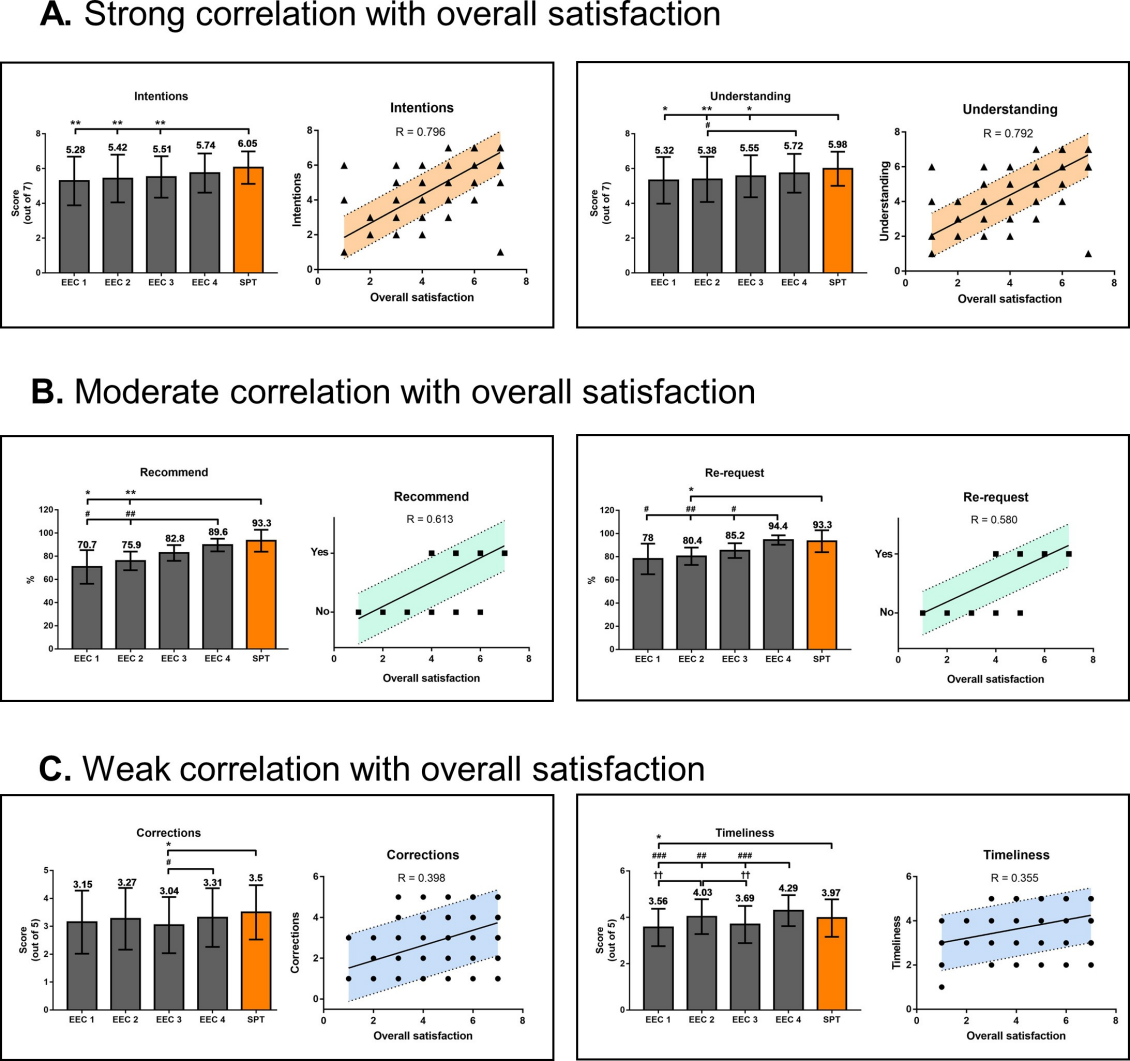
Specific question scores. Scores of specific questions (items #2 –#7 in Table 1) from blind survey results. Questions that showed (A) strong, (B) moderate, and (C) weak correlation with overall satisfaction score. Column bar graphs show the mean scores of each question (numbers on top of the bars); error bars indicate 95% confidence range in “Re-request” and “Recommend,” and standard deviation from mean in others. Scatter plots show the correlations between overall satisfaction and the specific questions. Trend between two variables are indicated by the solid lines. Shaded areas between dotted lines indicate 95% confidence range. * *P* < 0.05 vs. SPT, # *P* < 0.05 vs. EEC 4; **, ##, †† *P* < 0.01 vs. SPT, EEC 4, and EEC 2, respectively; ##, *P* < 0.001 vs. EEC 4; **** *P* < 0.0001 vs. SPT.

Fig 4 shows the results of the six specific questions that addressed issues such as whether the editing and comments were in line with the authors’ original intentions (Intentions) and how fast the turnaround was (Timeliness) (Table 1). Based on the survey response data as a whole, we tried to determine which of these factors were most closely associated with the overall satisfaction of editing service. As a result, we found that the degree to which the revisions and comments were in line with the authors’ original intentions (Intentions) was most strongly correlated with overall satisfaction (R = 0.796) (Fig 4A); in contrast, quicker return of the finished files (Timeliness) revealed the weakest correlations with overall satisfaction (R = 0.355) (Fig 4C).

### Changes in the author satisfaction scores and the proportion of unsatisfied responses

To determine whether the establishment of the SPT affected the author satisfaction of EECs, we gathered the survey response rate, the overall satisfaction scores, and the proportion of unsatisfied responses from the 18-months period prior to the study period (January 2016–June 2017) and compared them with the corresponding results from the study period (S6 Table). Notably, the overall survey response rate from the period before the establishment of the SPT was 9.0%, which was significantly lower than that from the period after the establishment of the SPT (53.2%, *P* < 0.0001). The overall satisfaction scores of the EECs as a whole did not show significant differences (*P* = 0.294). We also analyzed the proportion of unsatisfied responses in which the overall satisfaction scores were below 4 out of 7; as a result, we found that the proportion of unsatisfied responses were significantly lower in the “After” period (4.3%) than in the “Before” period (10.3%) (*P* = 0.009).

### Effect of internal quality control of external editing companies

Based on the author satisfaction survey results, the SPT performed regular quality control by informing the EECs of which editors received particularly positive or negative feedback so that those editors may be given priority or become delisted for AMC requests. Specifically, we noticed that EEC 2, a multinational company with a large number of freelance editors, had low overall satisfaction scores (4.93 out of 7) and occasional unfavorable reviews. Thus, we regularly informed EEC 2 of both notably good and bad reviews and provided comments on the specific issues raised by the authors. Through such a process of editor selection and feedback, the mean value of overall satisfaction scores gradually increased to 5.68 over the course of a year with a visible increasing trend (*P* for trend = 0.086) (S1 Fig).

### Changes in the number of research publications

From 2011 to 2017, the average number of quarterly research publications (i.e., articles and reviews) with AMC researchers as corresponding authors increased by approximately 34% (252.0 to 337.8) (S2A Fig). In 2018 (one year after the establishment of SPT), the average quarterly numbers of publications increased to 418 (orange bars, S2A Fig).

We also reanalyzed the publication trend data by employing segmented regression analysis of interrupted time series data as used in Fig 2B; this time, we regarded one year after the launch of the SPT as the time point of intervention (January 2018), considering the usual time lag between manuscript editing and acceptance. As a result, we observed statistically significant increases in the trend of research publications that listed AMC researchers as corresponding authors at one year after the establishment of the SPT (*P* for time after intervention = 0.00007) (S2B Fig).

## Discussion

Our experience of employing two professional editors to form the basis of an in-house editing team demonstrated that such a team was able to effectively manage > 2000 yearly editing requests while providing quality manuscript editing services themselves. The in-house editors received the highest mean scores in overall satisfaction in both blind and non-blind surveys, signifying how well the editorial service provided by the in-house editors was received. Importantly, the establishment of the in-house editing team was associated with a significant increase in the number of editing requests, and subsequently, the number of research publications from our medical center.

In order to facilitate the editing process and improve author satisfaction, in-house editing teams should selectively distribute editing requests according to the specific needs of each request: EECs may be better suited for handling requests for simple grammar checks or those that require quick turnaround time, while in-house editors may be more adept in handling more complex requests such as those requiring in-person consultations for content and organization of the manuscript. Such queries may be handled by the freelance editors of EECs as well, but as our manuscript editing workflow diagram shows, such a process (authors → in-house editing team → external company → freelance editor → external company → in-house editing team → authors) is considerably more arduous than receiving comments directly from the in-house editors.

In our study, the in-house editors received significantly higher overall satisfaction scores compared with all four EECs in the non-blind survey. As internal surveys may be inherently biased toward in-house teams, this was an expected outcome. Nevertheless, the in-house editors received the highest overall satisfaction score in the blind survey as well, which demonstrated significant differences with three of the four EECs, thus signifying their comparable competence. Also, the large difference between blind and non-blind survey scores of the SPT may be partly due to the fact that when editing non-blind manuscripts, the in-house editors were free to communicate with the authors prior to sending the final edited version, which enabled them to gain better understanding of the manuscript while editing. The in-house editors noted that such a process was crucial in providing higher quality editing services. Thus, the significantly higher scores of the SPT in the non-blind author satisfaction survey likely reflects the advantage of an in-house editing service compared with external editing.

Our author satisfaction survey was useful in evaluating the quality of editing service provided by the EECs and the in-house editors. Interestingly, the overall satisfaction showed weak correlation with the number of additional corrections the authors had to make or how quickly the manuscripts were returned. Instead, the conformity of edits and the level of understanding were strongly correlated with overall satisfaction. Thus, we suggest that questions on understanding and conformity of edits with author intentions be added to author satisfaction surveys, and that the editors should focus less on providing quick turnaround and more on investing time to provide thoughtful revisions based on deep understanding of the manuscript.

Our experience in establishing an in-house editing team may have broader relevance in English-speaking countries as well as non-native English-speaking countries. The numbers of international medical graduates (IMGs) have steadily increased in English-speaking countries such as the United States (US), Canada, the United Kingdom, and Australia, where IMGs now constitute more than a quarter of the total medical workforce [8–14]. As most of those IMGs are from non-native English-speaking countries, they may face a similar challenge in writing or presenting their research findings in English. As such, in-house editing departments in the US-based hospitals are well-received and have been correlated with improvements in writing and publication in general. Notably, the Department of Scientific Publications at The University of Texas MD Anderson Cancer Center developed a Scientific Writing Workshop for physicians with diverse language backgrounds, and reported that the workshop received robustly positive feedback [15]. In non-native English-speaking countries, Tokyo Medical University is known for its International Medical Communications Center, which has contributed to the increasing number and quality of research outputs by providing manuscript editing service and facilitation of medical communications [16]. Likewise, our experience at AMC has taught us that having a dedicated editing department greatly encourages non-native researchers who want to publish in English language journals. Thus, it would also behoove academic hospitals in English-speaking countries to establish their own editing departments to help their IMGs in terms of scientific publication.

Our study is limited by the fact that it was conducted in a single institution over a relatively short period of time (survey period: 18 months); such shortcomings may be at least partially compensated by the large number of survey responses (n = 936) and the randomized design of the surveys. Also, a portion of the author satisfaction survey data (21.6% of the blind survey data) had been collected according to 5-point scale scores and were later converted to 7-point scale scores in the final analysis, which may have introduced some bias in the analysis. However, the subgroup of survey responses that were collected in the 7-point scale scores did not show significantly different results compared with the main results, signifying that the addition of the converted 5-point scale scores did not introduce significant bias in the main analysis. Lastly, we were not able to track the submitted manuscripts to obtain data on the proportion of successfully published manuscripts or the time from editing request to publication of the manuscript. We could not gather such data because the title of the manuscripts often underwent changes in the finally published version and hindered their tracking. Also, the time from editing request to publication may not serve as a useful metric because journals have notable differences in the delay between acceptance and publication [17]. Changes in the mean impact factor of publications after the introduction of the in-house editing team would be a good metric and should be considered in future studies to better highlight the benefits of institutional support in English editing, while adjusting for the fact that impact factors are affected by the number of self-citations [18] and changes in the number of citable items [19].

As for the strength of the current study, the randomized design of the blind author satisfaction survey enabled us to obtain objective ratings on the quality of editing services provided by the EECs and the in-house editors. We believe that our suggested model of institutional support in English editing and the related findings would be directly applicable in medical centers in non-native English-speaking countries, which would include Asian countries as well as other countries with low proportions of English-speaking populations.

## Conclusions

The workflow of our in-house editing team depicts an implementable and practical model system for providing quality manuscript editing service by combining personalized editorial assistance by in-house editors and targeted quality control of external editing service providers. Importantly, the establishment of the in-house editing team was associated with hospital-wide increases in manuscript writing and publication. Thus, our model system may be adapted in academic hospitals to better address the editing needs of non-native English-speaking researchers and to encourage research publication.

## Supporting information

S1 Fig Overall satisfaction scores of EEC 2 over time. Changes in the overall satisfaction scores of EEC 2 according to blind survey results from 2018 Q1 to 2018 Q4. Numbers on top of the bars indicate the mean values, and numbers in parentheses indicate the number of survey results. Error bars indicate standard deviation from mean.
(TIF)

S2 Fig Trend in the number of research publications. Number of research publications with corresponding authors from Asan Medical Center from 2011 to 2018. (A) Quarterly number of publications from Asan Medical Center. Bars in orange indicate the period one year after the establishment of the Scientific Publications Team. Numbers on top of the bars indicate the actual number of scientific articles published during the respective quarters. (B) Trend in the number of research publications. Asterisks (***) indicate a significant (*P* for time after intervention = 0.0007) difference in the slope of research publication trend at one year after the intervention (establishment of the Scientific Publications Team).
(TIF)

S1 Table English editing request form. (DOCX)

S2 Table Details of the external editing companies (EECs). (DOCX)

S3 Table Author satisfaction survey response rates. (DOCX)

S4 Table 7-point + 5-point scale scores vs. 7-point scale scores only. (DOCX)

S5 Table Overall satisfaction score comparison in non-blind surveys between random and specific assignments. (DOCX)

S6 Table Comparison of author satisfaction responses between before and after the introduction of the Scientific Publications Team. (DOCX)

S1 File Raw data for Figs 2, 3 and 4, S1 Fig and S2 Fig. (XLSX)
