# Correction: Effects of providing manuscript editing through a combination of in-house and external editing services in an academic hospital

**DOI:** 10.1371/journal.pone.0232642

**Published:** 2020-04-28

**Authors:** Joon Seo Lim, Vanessa Topping, Ji Sung Lee, Keenan D. Bailey, Sung-Han Kim, Tae Won Kim

After publication of this article [1], concerns were raised about the relative scarcity regarding the overview of relevant literature supporting the study, up-to-date characterization of the field of academic editing, description of the editing services provided, and potential limitations of the current study.

**Overview of relevant literature:** The in-house editors at the SPT in Asan Medical Center are “author’s editors” as opposed to journal editors, and the concept of in-house author’s editors in universities and research centers has been previously described [2, 3]. Specifically, the establishment and management of in-house editorial teams in academic hospitals have also been reported [4, 5, 6, 7].

**Up-to-date characterization of the field of academic editing:** As of 2007, a total of 11 US-based medical schools report having in-house department/team dedicated to manuscript editing [6], a figure which had declined over the past few decades. Some academic hospitals in non-native English-speaking countries also have in-house editing teams [7, 8, 9].

**Description of the editing services provided at Asan Medical Center:** The approximate number of researchers at Asan Medical Center is 2000 (medical doctors, researchers, etc.). The annual number of editing requests is increasing and reached 2093 in 2018 (Fig 2A); each in-house editor of the SPT undertakes around 160 editing requests per year. The in-house editors either has a Master’s or a Doctorate degree in Biomedical research. Prior professional experience in academic editing was not a prerequisite for joining the in-house editing team. The in-house editors received on-the-job training during the first 6 months by (1) studying good and bad examples of editing, (2) editing a manuscript and comparing it to the version edited by an experienced in-house editor, (3) editing a manuscript, which is further edited by an experienced in-house editor, and studying the further edits. One of the in-house editors has received certifications from the BELS organization and the KCSE (Korean Council of Science Editors). The in-house editors dedicate approximately 8 hours per day editing manuscripts, part of which is irregularly devoted to discussion among the editors and one-on-one consultation with the researchers at Asan Medical Center. The median turnaround time for manuscripts is approximately 4 workdays for in-house editing and 3.5 workdays for external editing. In the majority of cases, manuscripts receive a single round of editing and are deemed ready for submission by the authors; additional rounds of editing are provided if the authors raise valid concerns regarding the quality of editing. The authors are free to acknowledge the external editing services in the published articles, and are encouraged to denote the editorial assistance of in-house editors if they were satisfied with the edits. Unless requested otherwise, the level of editing done by in-house editors is either substantive editing or didactic editing, in which the editors provide suggestions on word choice, sentence structure, scientific content, consistency, and logical flow via the Comment function in Microsoft Word; in case of one-on-one consultations, developmental editing was offered as well—these terminologies are explained in detail elsewhere [9]. To avoid ghost writing, the in-house editors do not add substantially new sentences or any content on their own.

**Fig 2:**
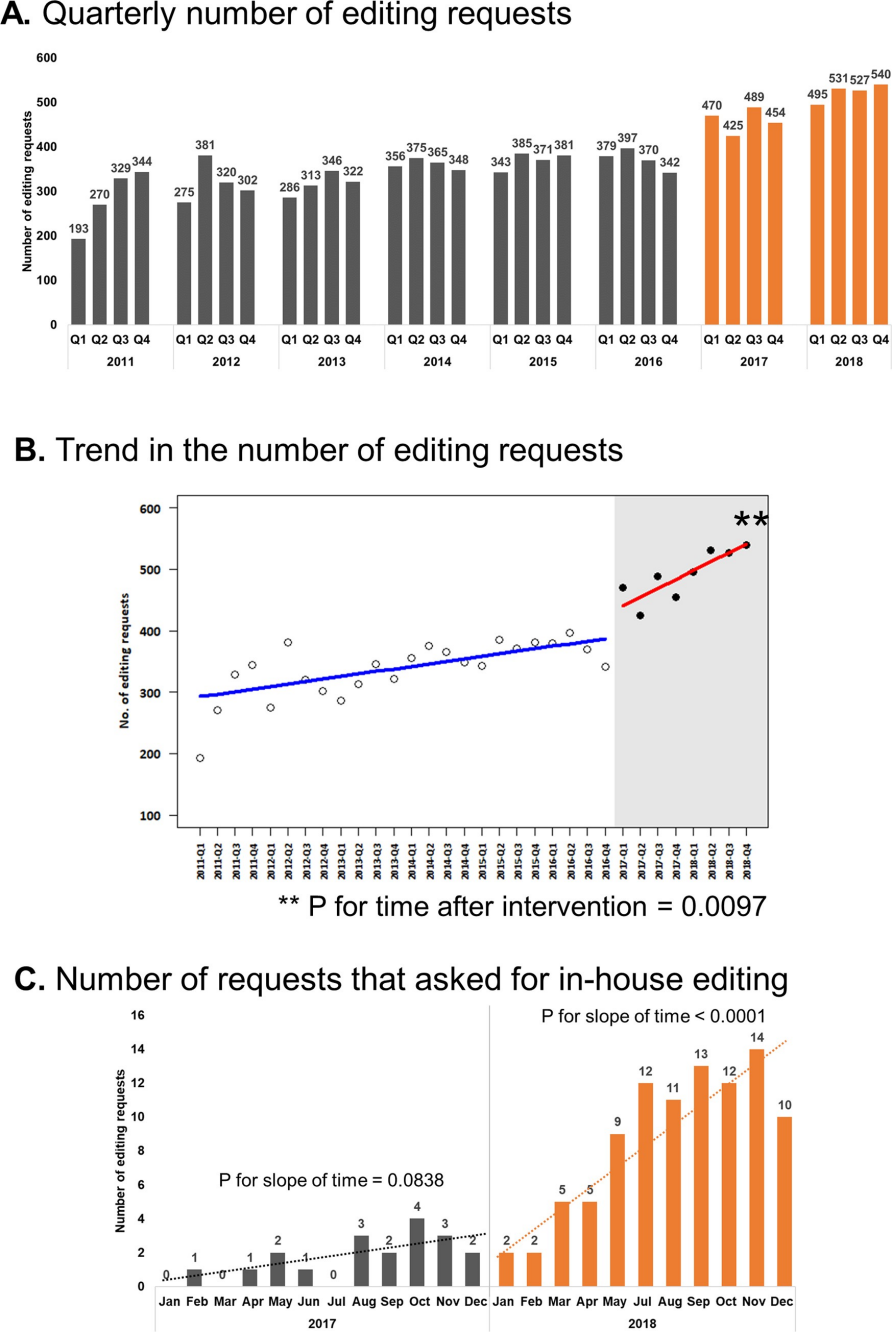
Trend in the number of editing requests. Trend of editing requests from AMC researchers from 2011 Q1 to 2018 Q4. (A) Quarterly number of editing requests. Numbers on top of the bars indicate the actual number of editing requests made during the respective quarters. Bars in orange indicate the period after which the Scientific Publications Team was established. (B) Trend in the number of editing requests. Asterisks (**) indicates a significant (P = 0.0097) difference in the slope of the editing request trend after intervention (establishment of the Scientific Publications Team; January 2017). (C) Number of requests that asked for in-house editing. Numbers on top of the bars indicate the actual number of editing requests that specifically asked for one of the in-house editors. Data from June 2018 was omitted because the in-house editors were on leave for most of the period, during which the number of editing requests was 2.

**Potential limitations:** Our study is limited in that it did not include a qualitative assessment of the impact of manuscript editing on the quality of research communication and publication process. Particularly, the impact of editing on both the authors and readers should be evaluated, which would require qualitative surveys or in-depth interviews. Also, following the suggestion of a reviewer during the peer review process, we added in the sixth paragraph of the Discussion sectionthat evaluating the “changes in the mean impact factor of publications after the introduction of the in-house editing team would be a good metric and should be considered in future studies.” Realizing the shortcomings of journal impact factors, we complemented the suggestion by acknowledging that impact factors are readily affected by the number of self-citations and changes in the number of citable items; even so, our suggestion may have been overreaching considering that journal impact factors are regarded to have more fundamental limitations that hinder their use as a sound metric for the quality of individual articles [10].
