# Supplementary figures and images for: Effects of providing manuscript editing through a combination of in-house and external editing services in an academic hospital
Source: PLoS One. 2019 Jul 9;14(7):e0219567. doi: 10.1371/journal.pone.0219567 (PMC6615627; doi:10.1371/journal.pone.0219567)

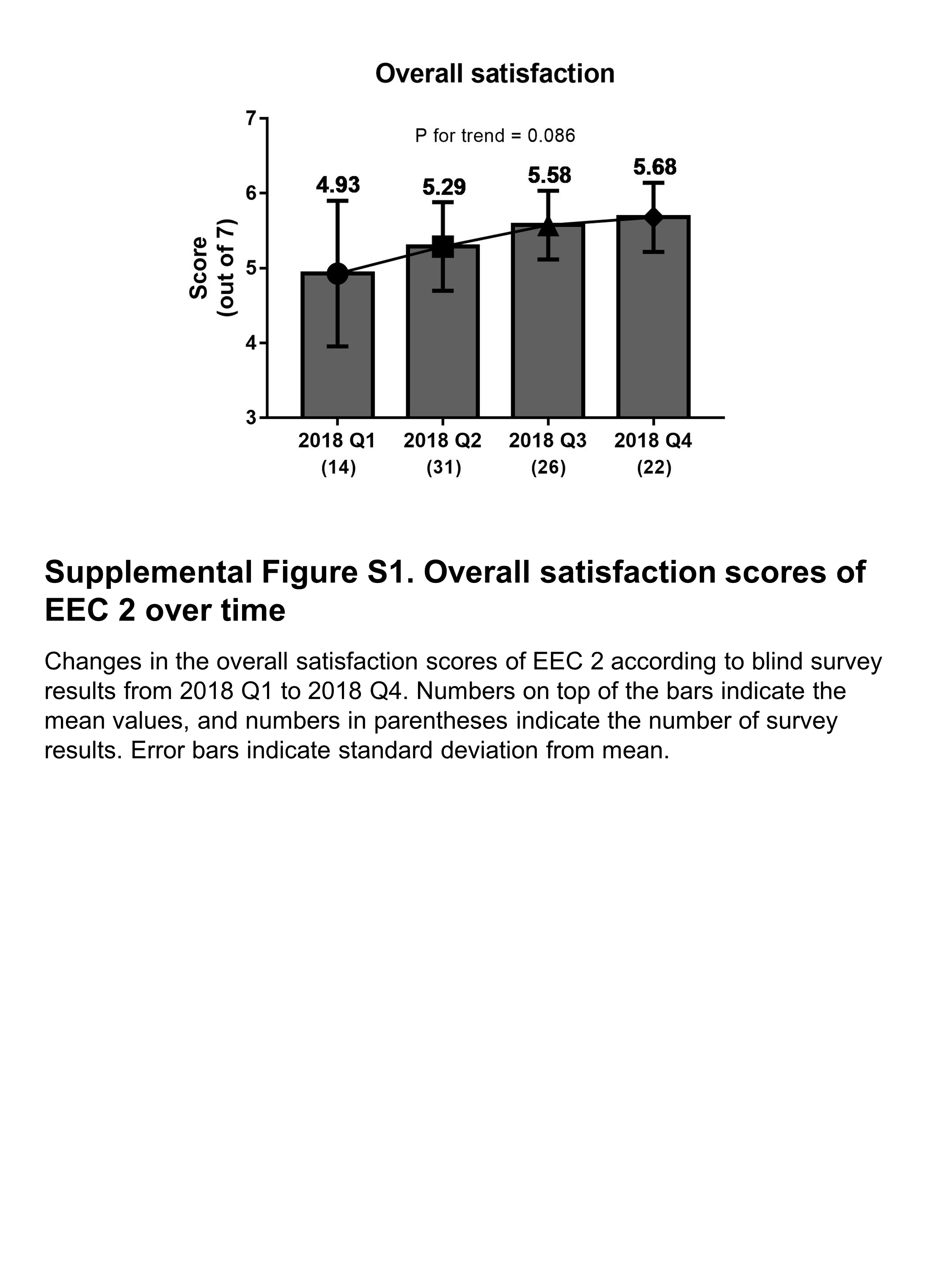

Supplement: S1 Fig — Changes in the overall satisfaction scores of EEC 2 according to blind survey results from 2018 Q1 to 2018 Q4. Numbers on top of the bars indicate the mean values, and numbers in parentheses indicate the number of survey results. Error bars indicate standard deviation from mean. (TIF) [file pone.0219567.s001.tif]

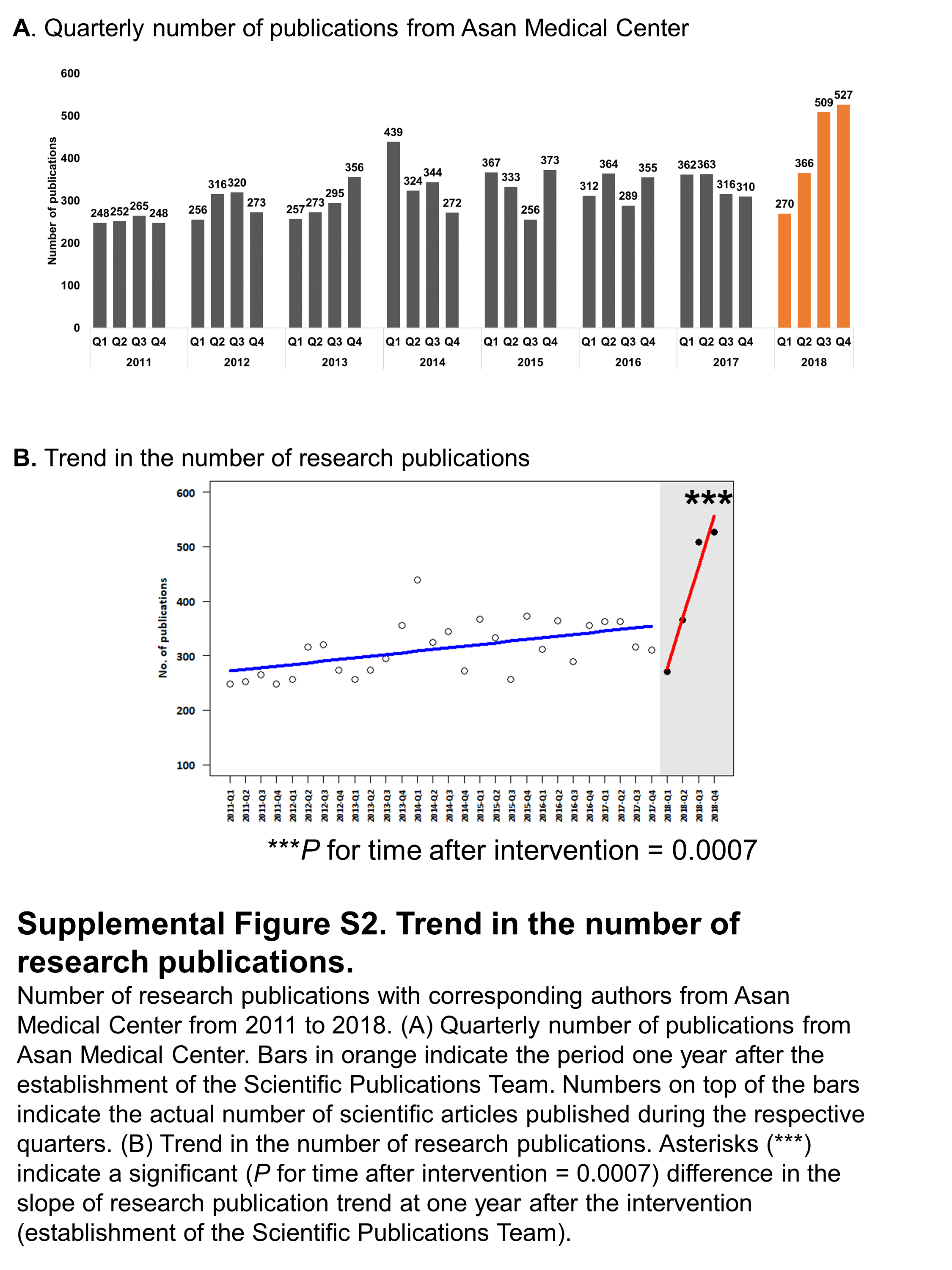

Supplement: S2 Fig — Number of research publications with corresponding authors from Asan Medical Center from 2011 to 2018. (A) Quarterly number of publications from Asan Medical Center. Bars in orange indicate the period one year after the establishment of the Scientific Publications Team. Numbers on top of the bars indicate the actual number of scientific articles published during the respective quarters. (B) Trend in the number of research publications. Asterisks (***) indicate a significant (P for time after intervention = 0.0007) difference in the slope of research publication trend at one year after the intervention (establishment of the Scientific Publications Team). (TIF) [file pone.0219567.s002.tif]
