# Supplementary material for: Effects of providing manuscript editing through a combination of in-house and external editing services in an academic hospital
Source: PLoS One. 2019 Jul 9;14(7):e0219567. doi: 10.1371/journal.pone.0219567 (PMC6615627; doi:10.1371/journal.pone.0219567)
Supplement: S1 Table — (DOCX) [file pone.0219567.s003.docx]

**Supplemental Table 1. English editing request form**

| # | Content | Note |
| --- | --- | --- |
| 1 | **Date of request** | (Automatically generated) |
|  | **Corresponding author information** |  |
| 2 | Name |  |
| 3 | Department |  |
| 4 | Position |  |
| 5 | E-mail address |  |
| 6 | Hospital extension number |  |
| 7 | Mobile phone number |  |
|  | **Requester information** |  |
| 8 | Name |  |
| 9 | Department |  |
| 10 | Position |  |
| 11 | E-mail address |  |
| 12 | Hospital extension number |  |
| 13 | Mobile phone number |  |
| 14 | **Type of the document** | (Choose one) |
|  | Original manuscript (first draft) |  |
|  | Revised manuscript (whole) |  |
|  | Revised manuscript (highlighted) |  |
|  | Conference abstract |  |
|  | Oral presentation script |  |
|  | Other |  |
| 15 | **Title of the manuscript** |  |
| 16 | **Number of files uploaded** |  |
| 17 | **Target journal/conference** | (For manuscript formatting) |
| 18 | **Field of study** | (For designating editors) |
| 19 | **Spelling type** | (Choose one) |
|  | American |  |
|  | British |  |
|  | **Word limit** | (Optional) |
| 21 | Abstract |  |
| 22 | Main text |  |
| 23 | **Notes** | Desired return date,  editing certificate, etc. |
