# Supplementary material for: Effects of providing manuscript editing through a combination of in-house and external editing services in an academic hospital
Source: PLoS One. 2019 Jul 9;14(7):e0219567. doi: 10.1371/journal.pone.0219567 (PMC6615627; doi:10.1371/journal.pone.0219567)
Supplement: S2 Table — (DOCX) [file pone.0219567.s004.docx]

**Supplementary Table S2.** **Details of the external editing companies (EECs)**

| **Company** | **Selection** | **Pricing** | **Editors’ profiles** | | | |
| --- | --- | --- | --- | --- | --- | --- |
|  |  |  | **Type** | **Number** | **Nationality** | **Credential** |
| EEC 1 | Recommendation by scientific journals  (e.g., Journal of Clinical Oncology, PNAS, Gastroenterology) | Word count | Freelance + in-house | ~10 | USA | MS, PhD |
| EEC 2 |  |  |  | ~1000 | USA, UK, Canada, Australia | BA, MS, PhD |
| EEC 3 |  |  |  | ~30 | USA, UK | PhD, MD |
| EEC 4 |  |  |  | ~800 | USA, UK, India, Canada, Australia | BA, MS, PhD |
