# Supplementary material for: Effects of providing manuscript editing through a combination of in-house and external editing services in an academic hospital
Source: PLoS One. 2019 Jul 9;14(7):e0219567. doi: 10.1371/journal.pone.0219567 (PMC6615627; doi:10.1371/journal.pone.0219567)
Supplement: S3 Table — (DOCX) [file pone.0219567.s005.docx]

**Supplementary Table S3. Author satisfaction survey response rates**

| **Company** | **Blind survey (n = 758)** | | | **Non-blind survey (n = 951)** | | |
| --- | --- | --- | --- | --- | --- | --- |
|  | **Response (n)** | | **Response rate (%)*** | **Response (n)** | | **Response rate (%)^#^** |
|  | **Yes** | **No** |  | **Yes** | **No** |  |
| EEC 1 | 41 | 33 | 55.4 | 63 | 51 | 55.3 |
| EEC 2 | 112 | 112 | 50.0 | 104 | 95 | 52.3 |
| EEC 3 | 122 | 79 | 60.7 | 79 | 81 | 49.4 |
| EEC 4 | 125 | 84 | 59.8 | 172 | 167 | 50.7 |
| SPT | 30 | 20 | 60.0 | 88 | 51 | 63.3 |
| Total | 430 | 328 | 56.7 | 506 | 445 | 53.2 |

Chi-square test: **P* = 0.1649, ^#^*P* = 0.0838
