# Supplementary material for: Effects of providing manuscript editing through a combination of in-house and external editing services in an academic hospital
Source: PLoS One. 2019 Jul 9;14(7):e0219567. doi: 10.1371/journal.pone.0219567 (PMC6615627; doi:10.1371/journal.pone.0219567)
Supplement: S4 Table — (DOCX) [file pone.0219567.s006.docx]

**Supplementary Table S4. 7-point + 5-point scale scores vs. 7-point scale scores only.**

|  | **Overall satisfaction score (blind survey)** | | | | |
| --- | --- | --- | --- | --- | --- |
|  | **7-point + 5-point scores**  **(Fig. 3A)** | | **7-point scores only** | | ***P* value**  **(mean)** |
|  | **n** | **Mean** | **n** | **Mean** |  |
| EEC 1 | 41 | 5.24 | 25 | 5.12 | 0.75 |
| EEC 2 | 112 | 5.40 | 87 | 5.39 | 0.97 |
| EEC 3 | 122 | 5.53 | 95 | 5.52 | 0.94 |
| EEC 4 | 125 | 5.76 | 106 | 5.80 | 0.78 |
| SPT | 30 | 5.97 | 24 | 6.08 | 0.66 |
| Total | 430 | 5.57 | 337 | 5.58 | 0.84 |
