# Supplementary material for: Effects of providing manuscript editing through a combination of in-house and external editing services in an academic hospital
Source: PLoS One. 2019 Jul 9;14(7):e0219567. doi: 10.1371/journal.pone.0219567 (PMC6615627; doi:10.1371/journal.pone.0219567)
Supplement: S5 Table — (DOCX) [file pone.0219567.s007.docx]

**Supplementary Table S5.** **Overall satisfaction score comparison in non-blind surveys between random and specific assignments**

|  | **Requests (n)** | | **Overall satisfaction score** | | |
| --- | --- | --- | --- | --- | --- |
| **Company** | **Random** | **Specific** | **Random** | **Specific** | ***P* value** |
| EEC 1 | 58 | 5 | 5.51 | 6.10 | 0.08 |
| EEC 2 | 73 | 31 | 5.61 | 5.85 | 0.31 |
| EEC 3 | 38 | 41 | 5.39 | 5.76 | 0.08 |
| EEC 4 | 119 | 53 | 6.02 | 5.89 | 0.50 |
| Total | 288 | 130 | 5.73 | 5.85 | 0.31 |
