# Supplementary material for: Effects of providing manuscript editing through a combination of in-house and external editing services in an academic hospital
Source: PLoS One. 2019 Jul 9;14(7):e0219567. doi: 10.1371/journal.pone.0219567 (PMC6615627; doi:10.1371/journal.pone.0219567)
Supplement: S6 Table — (DOCX) [file pone.0219567.s008.docx]

**Supplementary Table S6. Comparison of author satisfaction responses between before and after the introduction of the Scientific Publications Team.**

|  | **Response (n)** | | **Response rate (%)** | | | **Overall satisfaction (out of 7)** | | | | **Unsatisfied (%)** | | |  |
| --- | --- | --- | --- | --- | --- | --- | --- | --- | --- | --- | --- | --- | --- |
| **Company** | **Before** | **After** | **Before** | **After** | ***P* value** | **Before*** | **After** | ***P* value** | **Before** | | **After** | ***P* value** | |
| EEC 1 | 61 | 63 | 9.5 | 55.3 | **<0.0001** | 5.86 | 5.56 | 0.185 | 9.8 | | 6.3 | 0.526 | |
| EEC 2 | 59 | 104 | 8.9 | 52.3 | **<0.0001** | 5.73 | 5.68 | 0.827 | 15.3 | | 3.8 | **0.015** | |
| EEC 3 | 15 | 79 | 9.7 | 49.4 | **<0.0001** | 6.46 | 5.58 | **0.049** | 6.7 | | 2.5 | 0.410 | |
| EEC 4 | 50 | 172 | 8.5 | 50.7 | **<0.0001** | 5.93 | 5.98 | 0.822 | 6.0 | | 4.7 | 0.714 | |
| Total | 185 | 418 | 9.0 | 53.2 | **<0.0001** | 5.89 | 5.77 | 0.294 | 10.3 | | 4.3 | **0.009** | |

*Converted from 5-scale to 7-scale. Statically significant p values are shown in **bold**.
